# The Predictive Role of Immune Related Subgroup Classification in Immune Checkpoint Blockade Therapy for Lung Adenocarcinoma

**DOI:** 10.3389/fgene.2021.771830

**Published:** 2021-10-15

**Authors:** Xiaozhou Yu, Ziyang Wang, Yiwen Chen, Guotao Yin, Jianjing Liu, Wei Chen, Lei Zhu, Wengui Xu, Xiaofeng Li

**Affiliations:** ^1^ Department of Molecular Imaging and Nuclear Medicine, Tianjin Medical University Cancer Institute and Hospital, National Clinical Research Center for Cancer, Tianjin, China; ^2^ Key Laboratory of Cancer Prevention and Therapy, Tianjin, China; ^3^ Tianjin’s Clinical Research Center for Cancer, Tianjin, China; ^4^ Department of Molecular Imaging and Nuclear Medicine, Tianjin Cancer Hospital Airport Hospital, Tianjin, China

**Keywords:** lung adenocarcinoma, immune related subgroups, immune checkpoint blockade therapy, transcriptomics analysis, glucometabolic reprogramming

## Abstract

**Background:** In lung adenocarcinoma (LUAD), the predictive role of immune-related subgroup classification in immune checkpoint blockade (ICB) therapy remains largely incomplete.

**Methods:** Transcriptomics analysis was performed to evaluate the association between immune landscape and ICB therapy in lung adenocarcinoma and the associated underlying mechanism. First, the least absolute shrinkage and selection operator (LASSO) algorithm and K-means algorithm were used to identify immune related subgroups for LUAD cohort from the Cancer Genome Atlas (TCGA) database (*n* = 572). Second, the immune associated signatures of the identified subgroups were characterized by evaluating the status of immune checkpoint associated genes and the immune cell infiltration. Then, potential responses to ICB therapy based on the aforementioned immune related subgroup classification were evaluated *via* tumor immune dysfunction and exclusion (TIDE) algorithm analysis, and survival analysis and further Cox proportional hazards regression analysis were also performed for LUAD. In the end, gene set enrichment analysis (GSEA) was performed to explore the metabolic mechanism potentially responsible for immune related subgroup clustering. Additionally, two LUAD cohorts from the Gene Expression Omnibus (GEO) database were used as validation cohort.

**Results:** A total of three immune related subgroups with different immune-associated signatures were identified for LUAD. Among them, subgroup 1 with higher infiltration scores for effector immune cells and immune checkpoint associated genes exhibited a potential response to IBC therapy and a better survival, whereas subgroup 3 with lower scores for immune checkpoint associated genes but higher infiltration scores for suppressive immune cells tended to be insensitive to ICB therapy and have an unfavorable prognosis. GSEA revealed that the status of glucometabolic reprogramming in LUAD was potentially responsible for the immune-related subgroup classification.

**Conclusion:** In summary, immune related subgroup clustering based on distinct immune associated signatures will enable us to screen potentially responsive LUAD patients for ICB therapy before treatment, and the discovery of metabolism associated mechanism is beneficial to comprehensive therapeutic strategies making involving ICB therapy in combination with metabolism intervention for LUAD.

## Introduction

Lung cancer is one of the most common type of malignancies worldwide, and is the leading cause of cancer-related death among men and women globally (Siegel et al., 2021). Non-small cell lung cancer (NSCLC), which includes squamous cell carcinoma, adenocarcinoma and large cell carcinoma, accounts for more than 80% of all primary lung cancers (Kano et al., 2020). Within NSCLC, adenocarcinoma is the most common histological subtype (Zhang et al., 2020). Despite great improvements in LUAD treatment in recent decades, particularly molecular-targeted therapeutic strategies, such as tyrosine kinase inhibitors (TKIs) treatment targeting epidermal growth factor receptor (EGFR) and/or anaplastic lymphoma kinase (ALK) (Ge and Shi, 2015), the prognosis for LUAD patients remains poor with a 5-years survival rate of only 15% (Siegel et al., 2021). Fortunately, as an emerging therapeutic approach for tumor, immunotherapy, such as immune checkpoint blockade (ICB) therapy, is increasingly approved to be effective for LUAD (Huang et al., 2020a). Cytotoxic T-lymphocyte antigen 4 (CTLA-4) and programmed cell death protein 1/programmed cell death ligand 1 (PD-1/PD-L1) are crucial immune checkpoints to maintain homeostasis for immune response (Meyers and Banerji, 2020). Actually, attenuated anti-tumor immune response or induced immunosuppression in local tumor microenvironment (TME) partially result from excessive negative immune response mediated by immune checkpoints (Anichini et al., 2020). ICB therapy aims to enhance anti-tumor immune response by inhibiting detrimental immunosuppression induced by immune checkpoint in TME.

Owing to heterogeneity existing in LUAD and development of acquired resistance to ICB therapy, the overall performance of ICB therapy in clinical practice for LUAD is far from satisfactory (Pathak et al., 2020). As one of the most immunological cancer type, immunological surveillance, immunoediting and immune escape play a critical role in LUAD development and progression (Song et al., 2020). Screening for potentially responsive LUAD patients to ICB therapy before treatment by using an effective immunoligical biomarker is beneficial to remarkably improve the outcome of LUAD patients with ICB therapy (Wu et al., 2020). Tumor-infiltrating lymphocyte (TIL) score and PD-L1 expression in TME are previously suggested as potential biomarkers to select potentially sensitive subpopulation to ICB therapy prior to treatment and to predict survival for LUAD patients (Gascón et al., 2020; Jin et al., 2020; Hashemi et al., 2021). However, evaluations for the status of TIL and PD-L1 are currently non-standardized and limited by tissue samples availability. A comprehensive analysis of the immune associated signature in TME enable a further understanding of the interplay between local immune status and tumor immunotherapy responsiveness (Park et al., 2020; Wang et al., 2020).

**FIGURE 1:**
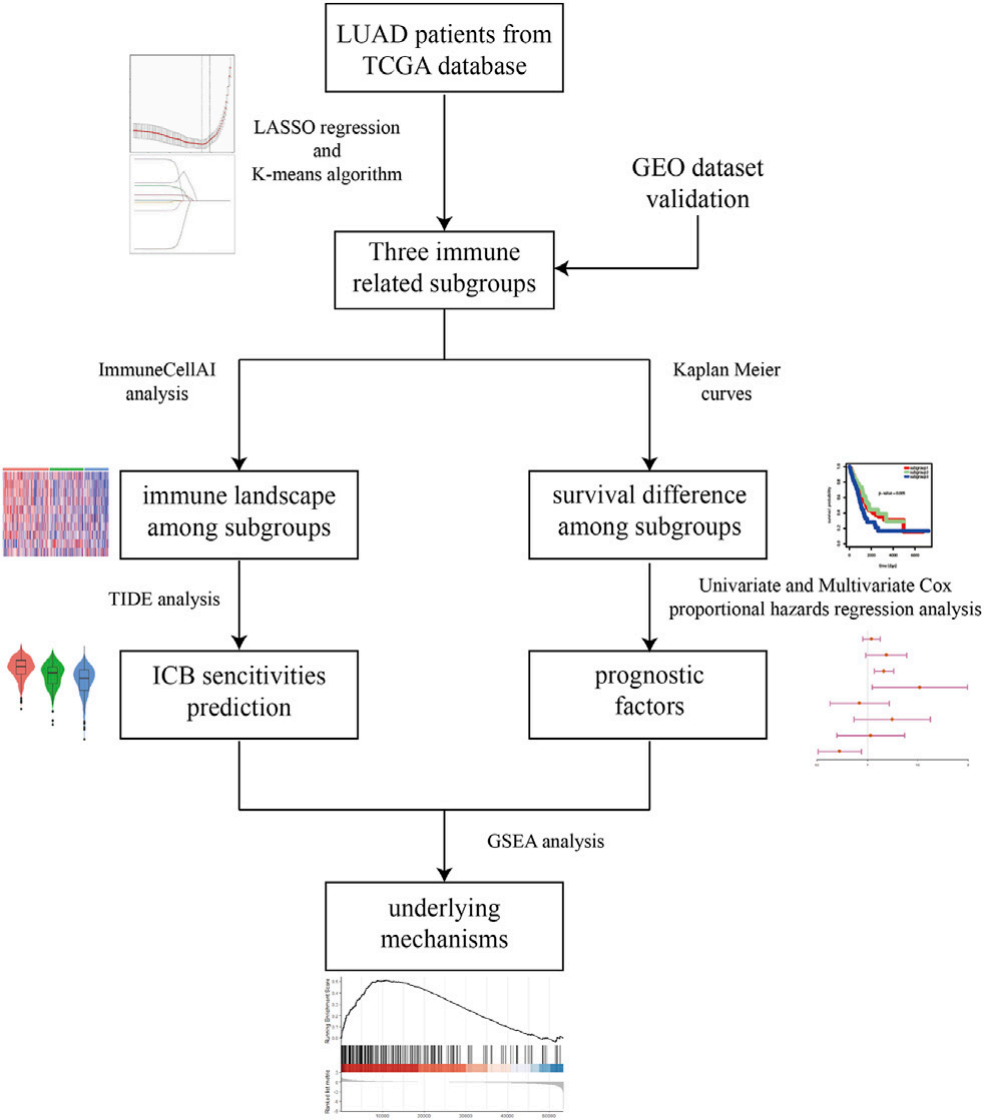
The workflow of this study. Briefly, immune related subgroup clustering was performed by using LASSO algorithm and K-means algorithm. After characterization of the immune associated signatures of the identified subgroups, TIDE algorithm analysis was performed to predict the potential sensitivities to ICB therapy. Meanwhile, survival analysis and further Cox proportional hazards regression analysis were also performed for LUAD. In the end, GSEA was performed to explore the metabolic mechanism potentially responsible for immune related subgroup clustering.

“Omics” techniques which are characterized by high-throughput interfaces are able to investigate complex biological systems in order to identify molecular signatures responsible for the complicated biological phenotype (Gillette et al., 2020; Lazarou et al., 2020). In the present investigation, bioinformatics analyses based on ribonucleic acid (RNA) sequencing (RNA-seq) data and clinical information from Cancer Genome Atlas (TCGA) database were performed to comprehensively explore the predictive role of immune associated signature in therapeutic responsiveness to ICB therapy for LUAD. First, immune related subgroup clustering was performed by using the least absolute shrinkage and selection operator (LASSO) algorithm and K-means algorithm. Second, the immune associated signatures of the identified subgroups were characterized by evaluating the status of immune checkpoint associated genes and the immune cells infiltration. Then, potential responses to ICB therapy were predicted via tumor immune dysfunction and exclusion (TIDE) algorithm analysis, and the relationship between the immune associated signature based on the aforementioned immune related subgroup classification and potential sensitivities to ICB therapy were determined. Additionally, survival analysis and further Cox proportional hazards regression analysis were also performed for LUAD, and gene set enrichment analysis (GSEA) was performed to explore the metabolic mechanism potentially responsible for immune related subgroup clustering. In the end, two microarray data sets from the Gene Expression Omnibus (GEO) database were used as validation cohorts in the study. The work flow of this study was shown in Figure 1.

Transcriptomics analysis of the association between immune associated signature and ICB therapy in LUAD not only explains for the heterogeneity in the reactivity to ICB therapy partially from an immunological perspective, but also provide potentially promising biomarker or target to direct sensitive LUAD patients screening prior to ICB therapy and combination therapy strategy making involving ICB therapy in combination with metabolism intervention.

## Materials and Methods

### Data Acquisition

The RNA-seq data sequenced on the Illumina RNA sequencing platform for LUAD samples from TCGA samples were download from the Cancer Genomics Browser of the University of California Santa Cruz (UCSC) Xena (https://xena.ucsc.edu/public) (Cline et al., 2013). Then, log2 (x+1) transformed HT-seq counts data and Fragments Per Kilobase Million (FPKM) data were selected for further analysis. The corresponding phenotype and survival information were also downloaded from the UCSC Xena. The latest gene ID annotation file (gencode.v32. annotation.gtf) was downloaded from the GENCODE database (http://www.gencodegenes.org) (Frankish et al., 2019) for Entrez gene ID and Ensembl gene ID transformation. Finally, after matching the TCGA sample ID in RNA-seq with the corresponding phenotype and survival information, a total of 572 LUAD samples in TCGA database were included in the study. Meanwhile, a total of 824 genes directly involved in immunological processes were collected using the Immunome database (Breuer et al., 2013). In addition, Microarray data for 398 LUAD samples in GSE72094 and 442 LUAD samples in GSE68465 were also acquired from the GEO database (https://www.ncbi.nlm.nih.gov/geo/). The corresponding gene chip annotation messages and clinical messages of this two data sets were downloaded using the R package GEOquery (Davis and Meltzer, 2007). The clinicopathological characteristics of LUAD patients from the training and validation sets were summarized in Table 1.

**TABLE 1:** Clinicopathological characteristics of LUAD patients from the training and validation sets.

Characteristics	TCGA	GSE68465	GSE72094
	Training set	Validation set	Validation set
Patient numbers	751	443	442
Age	65.2 ± 10.0	64.4 ± 10.1	69.2 ± 9.3
Gender	—	—	—
Male	342	223	202
Female	409	220	240
Tumor stages	—	—	—
Not reported	10	—	28
I	409	—	265
II	176	—	69
III	118	—	63
IV	38	—	17
Race	—	—	—
Not reported	70	129	45
Caucasian	581	295	399
African	84	12	13
Asian	16	7	3
Smoking history	—	—	—
Not reported	22	94	74
Never	108	49	33
Ever	621	300	335

### Data Preprocessing and Immune Related Subgroup Clustering

RNA-seq data and microarray data for LUAD from public database were first standardized for further analysis. “Combat” algorithm (Johnson et al., 2007) of R package sva (Leek et al., 2012) was employed to reduce the batch effect which may lead to deviations and bias to unrelated biological or scientific differences between subgroups (Leek et al., 2010). To filter out the missing values, intersective genes were selected from the TCGA cohort, GSE72094 cohort, GSE68465 cohort and Immunome database in the current study. Based on the expression of intersective genes for LUAD cohort from the TCGA database, LASSO algorithm and 10-fold cross validation method in R package glmnet (Friedman et al., 2010) were used to select the optimal gene set of the immune associated genes for immune related subgroup clustering. The total within sum of square and average silhouette width were calculated using R package factoextra to identify the best number of clustering. K-means algorithm, a classical unsupervised learning algorithm of artificial intelligence, was used for sample clustering in R software version 3.6.0 (https://www.r-project.org/) by 10 iterations with at least 30 samples for each subgroup. Moreover, consensus matrix analysis was performed in each data set to validate the clustering number, and consensus matrices were generated using the R package ConsensusClusterPlus (Wilkerson and Hayes, 2010). The principal component analysis (PCA) plot of the clustered samples were also drawn in the present study.

### Evaluation of Immune Cell Infiltration Scores and Immune Checkpoint Associated Genes Scores in Tumor Microenvironment as the Immune Associated Signature

Immune cell Abundance Identifier (ImmuCellAI) (Miao et al., 2020), a gene set signature-based method, was used to evaluate the infiltration scores of immune cells in the TME of LUAD. ImmuCellAI is capable of precisely estimating the abundance of 24 types of immune cell, including 18 T-cell subsets (CD4^+^, CD8^+^, CD4^+^ naïve, CD8^+^ naïve, central memory T (Tcm), effector memory T (Tem), Tr1, induced regulatory T cells (iTreg), natural regulatory T cells (nTreg), Th1, Th2, Th17, Follicular helper T cells (Tfh), cytotoxic T cells (Tc), mucosal-associated invariant T cells (MAIT), exhausted T cells (Tex), gamma delta T (γδ T), and natural killer T (NKT) cells) and six other important immune cells (B cells, macrophages, monocytes, neutrophils, dendritic cell (DC), and natural killer (NK) cells). In addition, it was reported that ImmuCellAI can estimate the abundance of immune cells with superior accuracy to other methods, especially on many T-cell subsets. Immune checkpoint associated genes, such as CTLA4, CD28, CD80, CD86, CD274 (PD-L1) and PD-1 (PDCD1), were selected from previous relevant studies focusing on the correlation between these genes and LUAD development, progression and prognosis.

### Prediction of Potential Sensitivity to Immune Checkpoint Blockades Therapy for Lung Adenocarcinoma Patients Based on Immune Related Subgroup Classification

Tumor immune dysfunction and exclusion (TIDE) algorithm (Fu et al., 2020) was used to calculate the potential possibility to respond to ICB therapy for LUAD patients based on immune related subgroup classification. Generally, TIDE analysis mainly consists of scores for TIDE, immune dysfunction, immune exclusion and several immune associated cells and effector molecules. Among which, negative score for TIDE suggests a lack of immune evasion phenotype. Meanwhile, T dysfunction score shows how a gene interacts with cytotoxic T cells to influence patient survival outcome, and the T cell exclusion score assesses the gene expression levels in immunosuppressive cell types that drive T cell exclusion. Scores for suppressive immune cells, such as cancer associated fibroblasts (CAF), myeloid-derived suppressor cell (MDSC), M2 macrophage indicate immune evasion or immunosuppression, suggesting a low possibility to respond to ICB therapy. Whereas, scores for effector immune cells, associated effector molecular and immune checkpoint associated genes, such as CD8^+^T cells, interferon-γ (IFN-γ) and PD-L1 (CD274) represent a potential sensitivity to ICB therapy. Additionally, immune related subgroup clustering, immune associated cells infiltration, immune checkpoint associated genes and clinicopathologic parameters, such as age, gender, pathological TNM stages, tumor stages in LUAD were also evaluated and analyzed between different immune related subgroups to perform a Cox proportional hazards regression analysis.

### Gene Set Enrichment Analysis (GSEA) to Explore the Underlying Mechanism Responsible for the Immune Related Subgroup Clustering of Lung Adenocarcinoma

GSEA is a bioinformatics analysis to determine whether a prior defined set of genes shows statistically significant and concordant differences between two groups (Sun et al., 2020). GSEA version 4.1.0, was used, the number of permutations was set to 1,000, and FDR <0.05 was the screening threshold. Given a close relationship between glucose metabolism reprogramming in tumor and anti-tumor immunomodulation, glucose metabolism process associated gene signatures, including the process of glycolysis, gluconeogenesis, tricarboxylic acid (TCA) cycle and oxidative phosphorylation (OXPHOS) in mitochondria were compared between the identified immune related subgroups (subgroup 1 vs subgroup 3) to explore the underlying mechanism responsible for the immune related subgroup clustering of LUAD.

### Statistical Analysis

The differences of immune associated signatures existed between immune related subgroups, such as the expression of immune check point genes and the infiltration scores of immune associated cells, were evaluated by using Kruskal-Wallis test. Before that, Shapiro-Wilk test and Tukey’s test were used to evaluate the status of normal distribution, and F test was used to perform homogeneity tests of variances. In addition, a survival analysis (overall survival) using Kaplan-Meier method was performed for LUAD patients, and the log-rank test was used to compare the differences of survival existed between the immune related subgroups aforementioned. Furthermore, univariate Cox proportional hazards regression analysis was performed to determine the correlation between survival and a variety of factors, including clinicopathologic parameters and immune associated signature factors. Afterwards, significantly associated factors were selected for further multivariate Cox proportional hazards regression analysis to determine independent risk factors. A *p*-value under 0.05 was considered to indicate a statistically significant difference. Data was analyzed using R software version 3.6.0. Multiple testing was corrected using the Benjamini-Hochberg’s false rediscovery rate (FDR).

## Results

### Immune-Associated Subgroup Clustering for Lung Adenocarcinoma From the Cancer Genome Atlas Database

**FIGURE 2:**
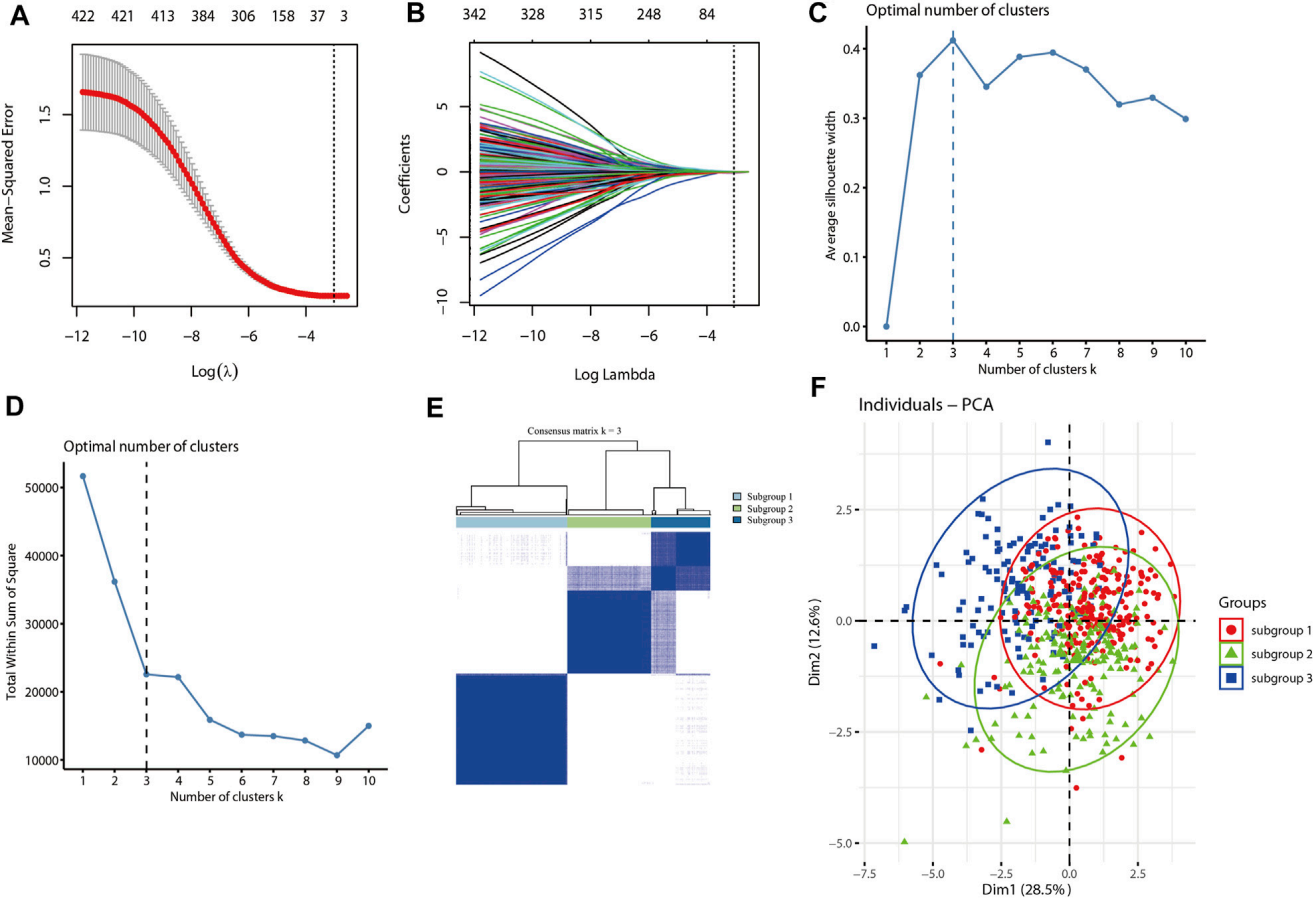
Immune associated subgroup clustering of LUAD by using the LASSO and K-means algorithm. **(A)** LASSO regression model with 10-cross validation was used to select the optimal λ (dash line) which have the minimum mean square error (red dots). **(B)** LASSO coefficient profiles of the selected subsets of immune associated genes at the optimal λ (grey line) for immune related subgroup clustering of LUAD. **(C)** The curve of average silhouette width under corresponding cluster number k, and the maximum of average silhouette width was achieved when k = 3. **(D)** The curve of total within sum of squared error curve under corresponding cluster number k, and it reached the “elbow point” when k = 3. **(E)** The consensus clustering of immune related subgroup of LUAD when k = 3. **(F)** The PCA plot of clustered samples in the LUAD, where samples in subgroup-1 (*n* = 252) are shown in red, subgroup-2 (*n* = 188) in green and subgroup-3 (*n* = 132) in blue.

The LASSO algorithm and 10-fold cross-validation were used to extract the optimal subsets of immune associated genes based on Immunome database for immune related subgroup clustering of LUAD cohort from TCGA database. As shown in Figure 2A, the optimal λ which have the minimum mean square error was selected by 10-fold cross validation. LASSO coefficient profile of the selected subsets of immune associated genes (*n* = 11) at the optimal λ for immune related subgroup clustering of LUAD was depicted in Figure 2B. To optimize the average silhouette width and the total within sum of square, the optimal number of clustering was set with k = 3 (Figures 2C,D). Based on this clustering, LUAD cohort (*n* = 572) from TCGA was divided into subgroup 1 (*n* = 252), subgroup2 (*n* = 188) and subgroup 3 (*n* = 132). The consensus matrix and the principal component analysis (PCA) plots of this immune related subgroup classification when k = 3 was shown in Figure 2E and Figure 2F, respectively. The results about this clustering were further validated in GSE72094 and GSE68465 data sets (Supplementary Figure S1).

### Characterization of Immune Associated Signature Based on Immune Related Subgroup Clustering of Lung Adenocarcinoma

**FIGURE 3:**
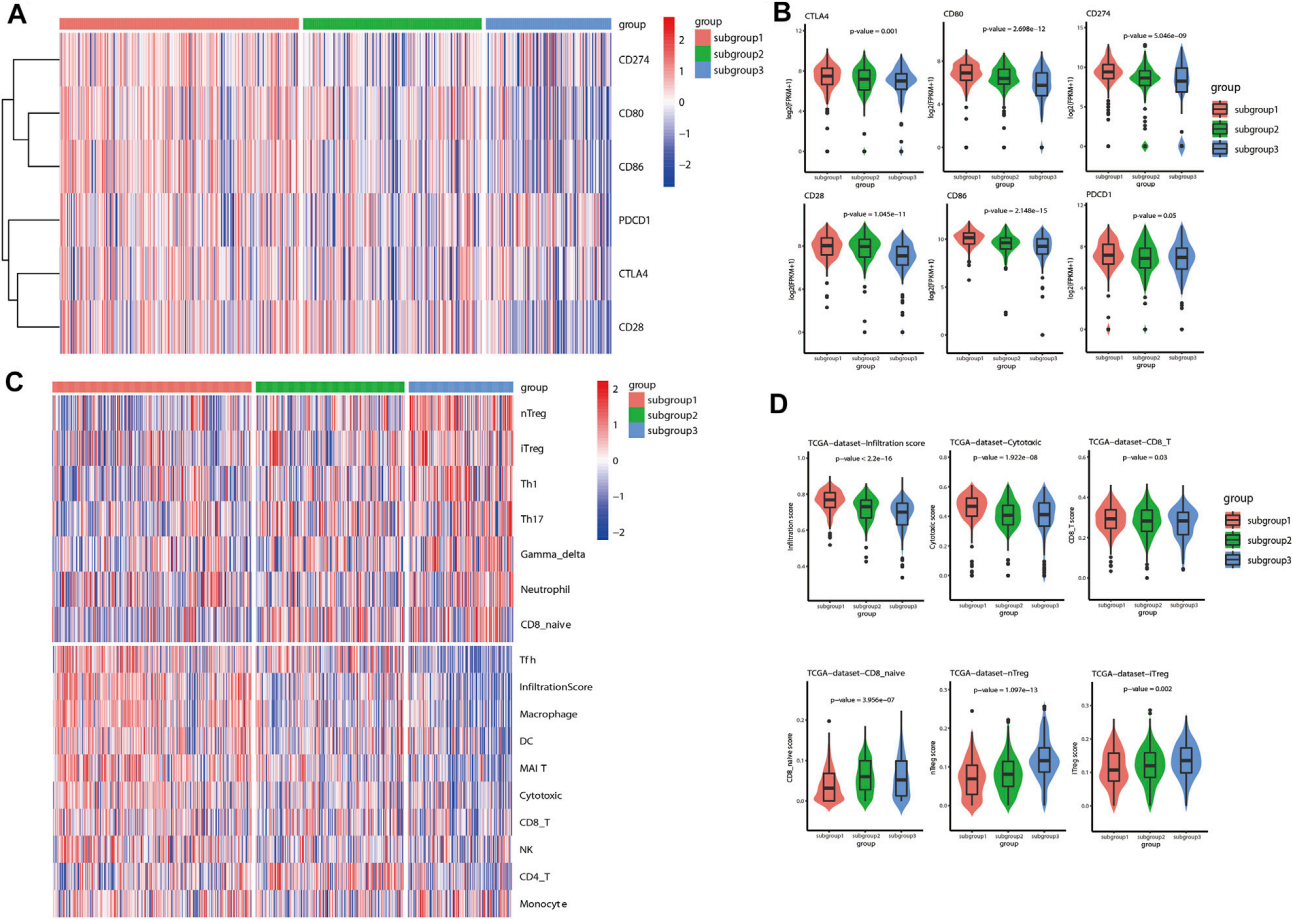
Characterization of immune associated signatures of the identified immune related subgroups of LUAD. **(A)** As shown in the gene expression heatmap, the levels of immune checkpoint associated genes, including CTLA4, CD28, CD80, CD86, CD274 (PD-L1) and PDCD1 (PD-1), were significantly different between the identified immune related subgroups of LUAD (*p* < 0.05). **(B)** Box plots were also shown to demonstrate the differences in each of these included immune checkpoint associated genes between the three subgroups. Generally, subgroup 1 tended to have higher expression levels of immune checkpoint associated genes in comparison with other subgroups (*p* < 0.05). **(C)** ImmuCellAI was used to evaluate the immune cell infiltration scores in the TME of LUAD. As shown in the heatmap, immune cell infiltration scores were found to be statistically different between the three immune related subgroups. The general infiltration score was remarkably higher in subgroup 1 in comparison with other subgroups, particularly with subgroup 3. **(D)** Box plots were also shown to indicate the differences between the three subgroups with regard to the infiltration scores of several representative immune cells. The infiltration scores of positive immune response, such as CD8^+^ cells and cytotoxic cells, were significantly higher in subgroup 1 than that in the other subgroups. Whereas, the infiltration scores of negative immune response, such as natural regulatory T cells (nTreg) and induced regulatory T cells (iTreg), were found to be statistically higher in subgroup 3 than that in other subgroups.

In the current investigation, immune checkpoint associated genes and immune cell infiltration scores were used to represent the immune associated signature of each of the immune related subgroups of LUAD. The status of immune checkpoint associated genes, such as CTLA4, CD28, CD80, CD86, PD-L1 (CD274) and PD-1 (PDCD1), were first evaluated for LUAD based on the aforementioned immune related subgroup clustering. As demonstrated in the heatmap (Figure 3A), the levels of these immune checkpoint associated genes were significantly different between the three subgroups (*p* < 0.05). Box plots were also used to show the differences in each of these immune checkpoint associated genes between the three subgroups (Figure 3B). Generally, subgroup 1 tended to have significantly higher expression levels of immune checkpoint associated genes in comparison with other subgroups, particularly with subgroup 3. Next, immune cell infiltration estimation was performed by using ImmuCellAI. As shown in Figure 3C, the general infiltration score was higher in subgroup1 in contrast with other subgroups, and a total of 16 immune cell infiltration scores were found to be statistically different between the three immune-related subgroups. In detail, the infiltration scores for effector immune cells, such as CD8^+^ cells and cytotoxic cells, were found to be statistically higher in subgroup 1 than that in other subgroups, whereas CD8 naive cell infiltration score was relatively lower in subgroup 1 compared to other subgroups. Meanwhile, the cell infiltration scores for suppressive immune cells, such as natural regulatory T cells (nTreg) and induced regulatory T cells (iTreg) were significantly higher in subgroup 3 than that in the other subgroups. (Figure 3D). Similar results with regard to the characterization of immune associated signature based on immune related subgroup clustering of LUAD were also validated in GSE72094 and GSE68465 data sets (Supplementary Figure S2 and Supplementary Figure S3).

### Estimation of Potential Sensitivity to Immune Checkpoint Blockades Therapy for Lung Adenocarcinoma Based on Immune Related Subgroup Clustering

**FIGURE 4:**
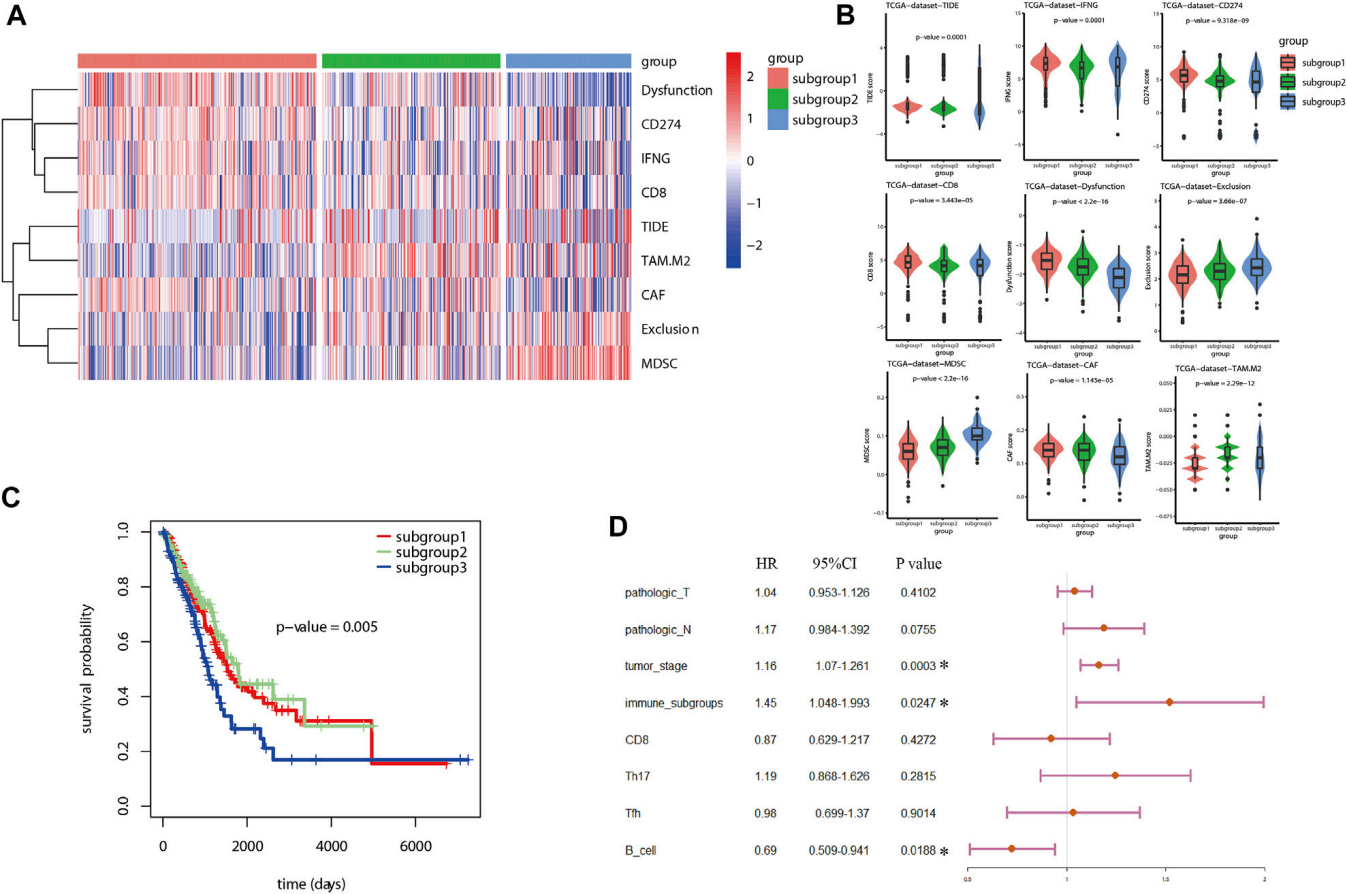
TIDE analysis and survival analysis for LUAD based on immune related subgroup clustering. **(A)** TIDE analysis was used to evaluate the potential sensitivity to ICB therapy for LUAD patients. As shown in the heatmap, the TIDE analysis associated scores were significantly different between the three subgroups (*p* < 0.05). **(B)** Based on the TIDE analysis, a higher potential sensitivity to ICB therapy was suggested for subgroup 1 which was with higher scores for TIDE, dysfunction, CD8^+^ cells and interferon-γ (IFN-γ), but with lower scores for exclusion, M2 macrophage and MDSC in comparison with that in subgroup 3. **(C)** Kaplan Meier analysis was performed to estimate the survival of LUAD. As shown, subgroup 3 tended to have an unfavorable prognosis in comparison with subgroup 1 and subgroup 2. **(D)** A total of 8 factors were included in further multivariate Cox proportional hazards regression analysis to identify independent risk factors for LUAD after an univariate Cox proportional hazards regression analysis. As shown in the forest plots, immune associated subgroup clustering, tumor stage and B cells infiltration were suggested as potential independent factors influencing overall survival (OS) of LUAD (*p* < 0.05).

Tumor immune dysfunction and exclusion (TIDE) algorithm was used to evaluate the potential sensitivity to ICB therapy for LUAD patients included in different immune related subgroups. As shown in the heatmap (Figure 4A), the TIDE analysis associated scores were significantly different between the three subgroups (*p* < 0.05). Based on the TIDE analysis, a higher potential sensitivity to ICB therapy was suggested for subgroup 1 which was with higher scores for TIDE, dysfunction, CD8^+^ cells, and interferon-γ (IFN-γ), but with lower scores for exclusion, M2 macrophage and MDSC in comparison with that in subgroup 3 (Figure 4B). Similarly, this TIDE analysis results were also validated in GSE72094 data sets (Supplementary Figure S3). GSE68465 data set was not used as validation cohort to perform TIDE analysis and survival analysis because of the lack of information for CD274.

### Survival Analysis and Cox Proportional Hazards Regression Analysis for Lung Adenocarcinoma

With regard to survival analysis for LUAD, the Kaplan Meier curves were drawn and the log-rank test was performed in this study. As demonstrated in Figure 4C, subgroup 3 tended to have an unfavorable prognosis in comparison with that of subgroup 1. Then, univariate Cox proportional hazards regression analysis was performed to identify the significant factors influencing the overall survival (OS) of LUAD. Among all the included factors, including the clinicopathologic parameters, immune checkpoint associated genes, immune cell infiltration scores, TIDE algorithm scores and immune related subgroup classification, a total of eight factors were proved to be significant risk factors influencing survival of LUAD (Table 2). Afterwards, all the eight factors were included in further multivariate Cox proportional hazards regression analysis to identify independent risk factors for LUAD. As shown in the forest plots (Figure 4D), immune related subgroup clustering, tumor stage and B cell infiltration were suggested as potential independent factors influencing OS of LUAD (*p* < 0.05). The results of survival analysis and Cox proportional hazards regression analysis in validation data set (GSE72094) were also shown in Supplementary Figure S4.

**TABLE 2:** Univariate Cox proportional hazards regression analysis of the prognostic factors for overall survival of LUAD.

Characteristics	HR	95% CI	P Value
Clinical features	—	—	—
Gender	1.05	0.79–1.41	0.72
Pathologic_T	1.18	1.09–1.27	< 0.01*
Pathologic_N	1.36	1.2–1.55	< 0.01*
Pathologic_M	0.98	0.9–1.07	0.68
Age	1.01	0.99–1.02	0.30
Tumor_stage	1.24	1.17–1.32	< 0.01*
Immune_subgroups	1.24	1.03–1.48	0.02*
Gene mutation	—	—	—
TP53	1.21	0.91–1.63	0.19
EGFR	1.4	0.94–2.1	0.10
KRAS	1.13	0.82–1.56	0.46
TIDE	—	—	—
TIDE	1.03	0.74–1.45	0.84
IFNG	1.13	0.84–1.52	0.42
CD274	1.16	0.85–1.57	0.35
CD8	0.72	0.54–0.97	0.02*
Dysfunction	0.85	0.63–1.14	0.26
Exclusion	1.31	0.98–1.77	0.07
CAF	1.11	0.83–1.49	0.47
TAM.M2	0.97	0.72–1.31	0.86
ImmuCellAI	—	—	—
CD4_naive	0.81	0.52–1.25	0.33
CD8_naive	0.9	0.67–1.21	0.49
Cytotoxic	0.92	0.69–1.23	0.58
Exhausted	0.92	0.68–1.23	0.55
Tr1	0.8	0.59–1.07	0.12
nTreg	1.18	0.88–1.58	0.26
iTreg	1	0.75–1.34	0.99
Th1	7.42	0.69–79.79	0.09
Th2	1.24	0.92–1.65	0.15
Th17	1.36	1.02–1.83	0.03*
Tfh	0.67	0.5–0.9	< 0.01*
Central_memory	1.11	0.83–1.5	0.47
Effector_memory	1.16	0.71–1.89	0.54
NKT	0.85	0.63–1.14	0.27
MAIT	0.93	0.7–1.25	0.63
DC	0.94	0.7–1.26	0.66
B_cell	0.6	0.45–0.82	< 0.01*
Monocyte	6.25	0.84–46.22	0.07
Macrophage	0.9	0.37–2.18	0.82
NK	1.04	0.78–1.39	0.78
Neutrophil	1.2	0.9–1.6	0.22
Gamma_delta	1	0.74–1.33	0.98
CD4_T	0.8	0.59–1.07	0.12
CD8_T	0.81	0.6–1.08	0.15
InfiltrationScore	0.82	0.62–1.1	0.19

Bold value indicates that the differences between groups were statistically significant.

### Potential Metabolism Associated Mechanism Responsible for Immune Related Subgroup Clustering of Lung Adenocarcinoma

**FIGURE 5:**
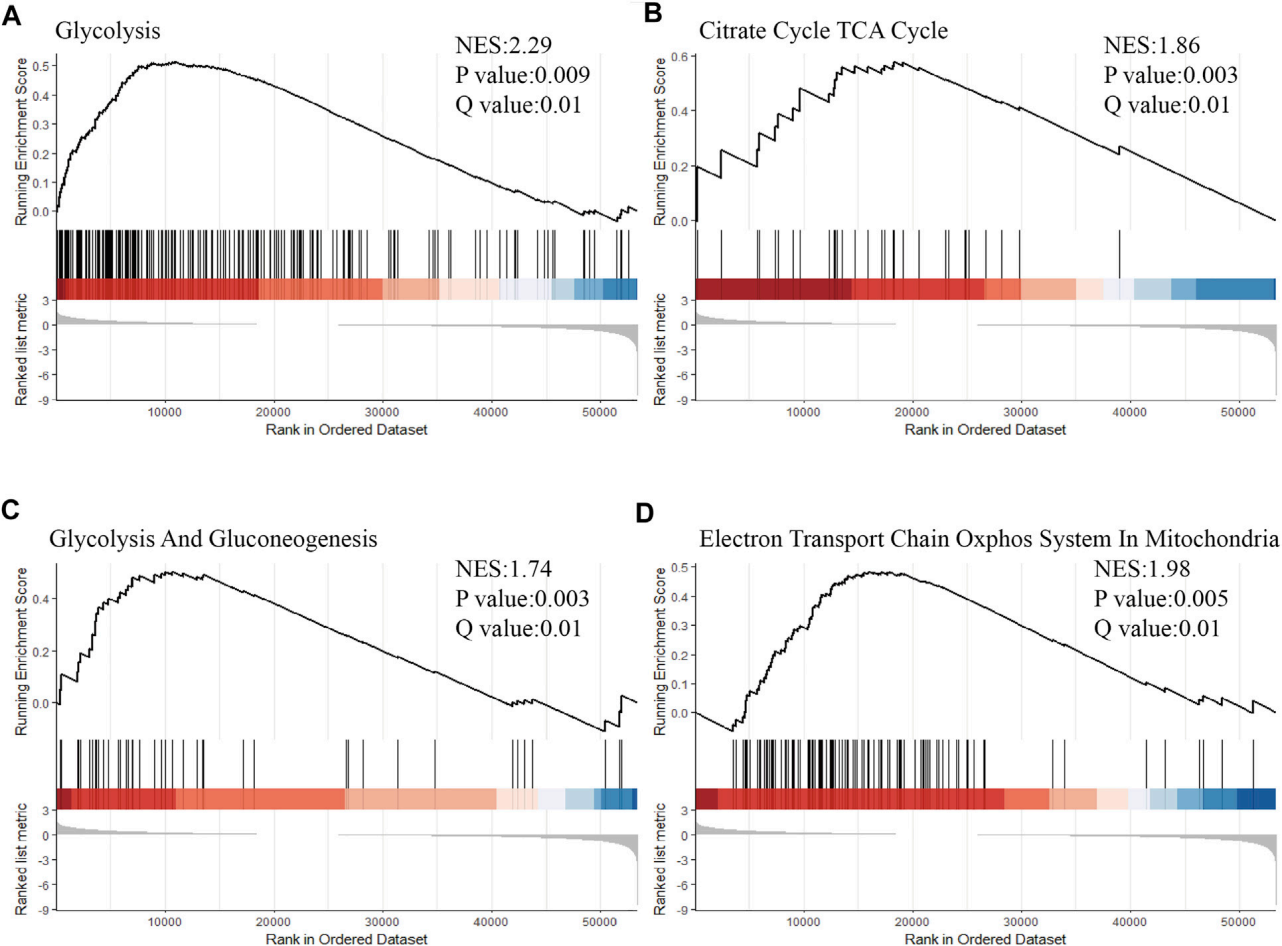
Glucose metabolic reprogramming was suggested as one of the underlying mechanisms for immune related subgroup clustering of LUAD. Based on the immune related subgroup clustering (subgroup 1 vs subgroup 3), GSEA was performed by using the gene sets significantly associated with glucose metabolism, including processes of **(A)** glycolysis (Normalized Enrichment Score (NES) = 2.29, *p* < 0.01, Q < 0.05), **(B)** tricarboxylic acid (TCA) cycle (NES = 1.86, *p* < 0.01, Q < 0.05), **(C)** gluconeogenesis (NES = 1.74, *p* < 0.01, Q < 0.05) and **(D)** oxidative phosphorylation (OXPHOS) in mitochondria (NES = 1.98, *p* < 0.01, Q < 0.05) (NES = 1.98, *p* < 0.01, Q < 0.05). FDR <0.05 was set as the screening threshold. An upward parabola indicated that the indicated process was enhanced in subgroup 1 in contrast with subgroup 3. The barcode plot indicates the position of the genes in each gene set; red and blue colors represent positive and negative Pearson’s correlation with subgroup classification.

Based on the immune related subgroup clustering (subgroup 1 vs subgroup 3), gene set enrichment analyses (GSEA) was performed on LUAD data set from the TCGA database using the gene sets significantly associated with glucose metabolism, including the process of glycolysis (Figure 5A), tricarboxylic acid (TCA) cycle (Figure 5B), gluconeogenesis (Figure 5C), oxidative phosphorylation (OXPHOS) in mitochondria (Figure 5D). FDR (Q value) < 0.05 was set as the screening threshold. As shown, the upward parabolas indicated that all the included processes of glucose metabolism was enhanced in subgroup 1 in contrast with that in subgroup 3. Glucose metabolic reprogramming was suggested as one of the underlying mechanisms for immune related subgroup clustering of LUAD. The results of GSEA analysis in validation data set (GSE72094 and GSE68465) were also shown in Supplementary Figure S5.

## Discussion

As an emerging therapeutic approach for malignancies, tumor immunotherapy, particularly for ICB therapy, is increasingly proved to be effective for LUAD patients (Huang et al., 2020a; Kano et al., 2020). However, a remarkable improvement in overall response rate and prognosis for LUAD patients is still not achieved due to the inherent intertumoral and intratumoral heterogeneity and the development of acquired resistance to ICB therapy (Jin et al., 2020; Song et al., 2020). To address this issue, a promising biomarker which is capable of predicting therapeutic efficiency before treatment is needed to screen potential responsive subpopulation prior to treatment and monitor the therapeutic efficiency during the process of treatment (Meyers and Banerji, 2020). Tumor-immune relationship plays an important role in tumor development and tumor progression, and tumor immune microenvironment (TIM) is widely accepted as a significant factor influencing therapeutic efficiency of ICB therapy (Song et al., 2020; Wang et al., 2020). Specifically, tumor-infiltrating lymphocytes (TILs) score (Gascón et al., 2020; Hashemi et al., 2021) and PD-L1 status (Wu et al., 2020) were previously suggested as potential biomarkers to be applied in clinical practice for LUAD. However, its translation from bench to bedside is largely limited by the dependence on tissue sample availability and the non-standardization for evaluation of TIL score and PD-L1 expression. Though previous studies tried to use immunophenotypic subtype classification based on immune signature to address this issue (Song et al., 2020; Wang et al., 2020; Xu et al., 2020), a systematic and comprehensive analysis (Seo et al., 2018; Zhang et al., 2020) is still required to determine the correlation between immune landscape based on immune related subgroup clustering and therapeutic reactivity to ICB therapy, and the underlying mechanism is of necessity to be explored (Huang et al., 2020b; Giannone et al., 2020). Previous studies from Chen YS. et al. (Xu et al., 2020) and Chen KX. et al. (Seo et al., 2018) performed immune related subgroup classification by using computational algorithms, however, an elaborated immune landscape characterization for distinct immune related subgroups were inadequate. Even though results from Xing Y. et al. (Song et al., 2020) and Kim Y. et al. (Xu et al., 2020) suggested a potential implication of immune subtype classification for ICB immunotherapy in lung cancer, a comprehensive analysis of the potential response to ICB immunotherapy for lung cancer, such as TIDE algorithm, was actually lacked. In the present investigation, we focused on both the elucidation of different immune signatures and prediction of potential response to ICB therapy for lung adenocarcinoma based on immune related subgroup clustering by using K-means algorithm, a classical unsupervised learning algorithm of artificial intelligence. More importantly, we conducted GSEA analysis to explore metabolism associated mechanism potentially responsible for immune related subgroup clustering of LUAD, particularly emphasized on the glucometabolic mechanism to shed light on comprehensive treatment strategy involving ICB immunotherapy in combination with glucose metabolism intervention.

Three distinct immune related subgroups were classified for LUAD in the current study based on RNA-seq data set from TCGA database (*n* = 572) by using a K-means algorithm. Among the classification, subgroup 1 was characterized by higher levels of immune checkpoint associated genes and higher cell infiltration scores for immune associated effector cells, and tended to be more sensitive to ICB therapy and have a favorable prognosis. Whereas, subgroup 3 with lower levels of immune checkpoint associated genes but higher cell infiltration scores for immune associated suppressive cells was found to be less responsive to ICB therapy and have a poor prognosis. Presumedly, subgroup 1 represented an immune-hot or with an immunocompetent TME with a higher infiltration score and an immunocompetent subtype which was possibly associated with a potential response to ICB therapy and a favorable prognosis. Whereas, subgroup 3 was considered as an immunodeficient or immunosuppressive landscape with a lower infiltration score or with an immunosuppressive subtype, suggesting a potential resistance to ICB therapy and an unfavorable prognosis. With respect to subgroup 2, a median subtype with a mixture of characteristics of subgroup 1 and subgroup 3 was considered. After Kaplan Meier analysis and Cox proportional hazards regression analysis, the immune related subgroup clustering was found to be an independent risk factor influencing the OS of LUAD patients. In the end, the GSEA analysis revealed that the metabolic reprogramming status in LUAD is potentially one of the underlying mechanisms for the distinct immune associated signatures based on the immune related subgroup clustering (Hensley et al., 2016; Faubert et al., 2017; Smolle et al., 2020). The enhanced glucose metabolism in subgroup 1 was consistent with the immune-hot landscape and a relatively immunocompetent subtype, whereas the decreased glucose metabolism in subgroup 3 suggested an immunodeficient landscape and/or an immunosuppressive subtype. Validation LUAD cohorts from external GEO database were also used to confirm the aforementioned results. To sum up, the present investigation provided a deep understanding of the interaction between tumor cells and surrounding immune cells (Kareva and Hahnfeldt, 2013; Speiser et al., 2016) and shed light on an improvement in ICB therapy or derived combination treatment for LUAD involving ICB therapy and metabolism intervention treatment.

As we know that, metabolic reprogramming and immunomodulation are two hallmarks of tumor (Hanahan and Weinberg, 2011). From a metabolic perspective, both tumorigenesis and immunoregulation are intricately associated with metabolic reprogramming. Specifically, the metabolic interplay between tumor cells and infiltrating immune cells significantly contributes to tumor progression and tumor immunosuppression. As reported previously, metabolic competition between tumor cells and surrounding immune cells (Chang et al., 2015) and an accumulation of a variety of metabolite caused by metabolic reprogramming (Feng et al., 2017) in TME are partially responsible for immune landscape remodeling. Even though improvement in ICB therapy for LUAD in recent decades, a potential marker for effective stratification of LUAD patients before treatment and a promising target for associated molecular targeted therapy in combination with ICB therapy are expected to bring out breakthrough to clinical management for LUAD. The heterogeneity in metabolism status of LUAD was previously described (Hensley et al., 2016) and further confirmed by metabonomics analysis by investigation from others (Lazarou et al., 2020; Zhao et al., 2020). Additionally, multi-omics analysis based on single cell sequencing data also recovered a close correlation between immune status and metabolic reprogramming (Kim et al., 2020; Xiao et al., 2020; Zhong et al., 2021). Therefore, ICB therapy combined with metabolism intervention is expected to improve the prospect of LUAD treatment.

In spite of the innovation and valuable results mentioned above with respect to this study, a few limitations existing in the current investigation is noteworthy. First, the TCGA database mainly comprises Caucasian population, while validation cohort from GEO database mostly consists of Asian patients, thus racial bias was not inevitable in this study. To attenuate this bias, two external validation cohorts from GEO database were used to validate the results. Then, as actual sensitivity to ICB therapy for LUAD was not available in this study because the clinical information regarding to ICB therapy was mostly not provided in TCGA and GEO databases, only potential reactivity to ICB therapy for LUAD was evaluated based on TIDE analysis. In the end, the correlation between immune associated signature and sensitivity to ICB therapy and underlying metabolic reprogramming-associated mechanism were not further validated by basic research *in vitro* and clinical investigation *in vivo*, which is what we aim to do in future.

## Conclusion

In the current investigation, a novel immune related subgroup clustering by an unsupervised learning model was identified for LUAD. Distinct immune associated landscape based on this clustering was significantly correlated with potential sensitivity to ICB therapy and prognosis for LUAD. GSEA analysis revealed that the heterogeneity in metabolic reprogramming is potentially one of the underlying mechanisms responsible for the correlation between immune landscape and potential reactivity to ICB therapy for LUAD. The immune related subgroup clustering based on the transcriptomics analysis will enable us to screen potentially responsive LUAD patients to ICB therapy. Additionally, metabolism intervention is a promising approach to improve the therapeutic efficiency of ICB therapy for LUAD.

## Data Availability

The original contributions presented in the study are included in the article/Supplementary Material, further inquiries can be directed to the corresponding authors.

## Glossary

ANN: artificial neural network
ALK: anaplastic lymphoma kinase
CTLA-4: cytotoxic T-lymphocyte antigen 4
CLP: common lymphoid progenitor
CAF: cancer-associated fibroblast
DC: dendritic cell
DBSCAN: density-based spatial clustering of applications with noise
EGFR: epidermal growth factor receptor
FDR: false rediscovery rate
GEO: Gene Expression Omnibus
GSEA: single-sample gene set enrichment analysis
HR: hazard ratio
ICBs: immune checkpoint blockades
IFN-γ: interferon-γ
ImmuCellAI: Immune cell abundance identifier
LASSO: least absolute shrinkage and selection operator
LUAD: lung adenocarcinoma
MAIT: mucosal-associated invariant T
MDSC: myeloid-derived suppressor cell
NK: natural killer
NKT: natural killer T
NSCLC: non-small cell lung cancer (NSCLC)
OS: Overall survival
OXPHOS: oxidative phosphorylation
PD-1/PD-L1: programmed cell death protein 1/programmed cell death ligand 1
PCA: principal components analysis
RNA-seq: RNA sequencing
SVM: support vector machine
TCA: tricarboxylic acid
Tc: cytotoxic T
TCGA: the Cancer Genome Atlas
Tex: exhausted T cells
TIDE: tumor immune dysfunction and exclusion
TIL: tumor-infiltrating lymphocyte
TIM: tumor immune microenvironment
TKIs: tyrosine kinase inhibitors
TME: tumor microenvironment
Treg: Regulatory T

## References

[B1] Anichini A. Perotti V. E. Sgambelluri F. Mortarini R. (2020). Immune Escape Mechanisms in Non Small Cell Lung Cancer. Cancers 12 (12), 3605. 10.3390/cancers12123605 PMC7761620 33276569

[B2] Breuer K. Foroushani A. K. Laird M. R. Chen C. Sribnaia A. Lo R. (2013). InnateDB: Systems Biology of Innate Immunity and Beyond-Rrecent Updates and Continuing Curation. Nucleic Acids Res. 41 (Database issue), D1228–D1233. 10.1093/nar/gks1147 23180781 PMC3531080

[B3] Chang C.-H. Qiu J. O’Sullivan D. Buck M. D. Noguchi T. Curtis J. D. (2015). Metabolic Competition in the Tumor Microenvironment Is a Driver of Cancer Progression. Cell 162 (6), 1229–1241. 10.1016/j.cell.2015.08.016 26321679 PMC4864363

[B4] Cline M. S. Craft B. Swatloski T. Goldman M. Ma S. Haussler D. (2013). Exploring TCGA Pan-Cancer Data at the UCSC Cancer Genomics Browser. Sci. Rep. 3, 2652. 10.1038/srep02652 24084870 PMC3788369

[B5] Davis S. Meltzer P. S. (2007). GEOquery: a Bridge between the Gene Expression Omnibus (GEO) and BioConductor. Bioinformatics 23 (14), 1846–1847. 10.1093/bioinformatics/btm254 17496320

[B6] Faubert B. Li K. Y. Cai L. Hensley C. T. Kim J. Zacharias L. G. (2017). Lactate Metabolism in Human Lung Tumors. Cell 171 (2), 358–371. 10.1016/j.cell.2017.09.019 28985563 PMC5684706

[B7] Feng J. Yang H. Zhang Y. Wei H. Zhu Z. Zhu B. (2017). Tumor Cell-Derived Lactate Induces TAZ-dependent Upregulation of PD-L1 through GPR81 in Human Lung Cancer Cells. Oncogene 36 (42), 5829–5839. 10.1038/onc.2017.188 28604752

[B8] Frankish A. Diekhans M. Ferreira A.-M. Johnson R. Jungreis I. Loveland J. (2019). GENCODE Reference Annotation for the Human and Mouse Genomes. Nucleic Acids Res. 47 (D1), D766–D773. 10.1093/nar/gky955 30357393 PMC6323946

[B9] Friedman J. Hastie T. Tibshirani R. (2010). Regularization Paths for Generalized Linear Models via Coordinate Descent. J. Stat. Softw. 33 (1), 1–22. 10.18637/jss.v033.i01 20808728 PMC2929880

[B10] Fu J. Li K. Zhang W. Wan C. Zhang J. Jiang P. (2020). Large-scale Public Data Reuse to Model Immunotherapy Response and Resistance. Genome Med. 12 (1), 21. 10.1186/s13073-020-0721-z 32102694 PMC7045518

[B11] Gascón M. Isla D. Cruellas M. Gálvez E. M. Lastra R. Ocáriz M. (2020). Intratumoral versus Circulating Lymphoid Cells as Predictive Biomarkers in Lung Cancer Patients Treated with Immune Checkpoint Inhibitors: Is the Easiest Path the Best One? Cells 9 (6), 1525. 10.3390/cells9061525 PMC7348938 32580514

[B12] Ge L. Shi R. (2015). Progress of EGFR-TKI and ALK/ROS1 Inhibitors in Advanced Non-small Cell Lung Cancer. Int. J. Clin. Exp. Med. 8 (7), 10330–10339. 26379824 PMC4565207

[B13] Giannone G. Ghisoni E. Genta S. Scotto G. Tuninetti V. Turinetto M. (2020). Immuno-Metabolism and Microenvironment in Cancer: Key Players for Immunotherapy. Int. J. Mol. Sci. 21 (12), 4414. 10.3390/ijms21124414 PMC7352562 32575899

[B14] Gillette M. A. Satpathy S. Cao S. Dhanasekaran S. M. Vasaikar S. V. Krug K. (2020). Proteogenomic Characterization Reveals Therapeutic Vulnerabilities in Lung Adenocarcinoma. Cell 182 (1), 200–225.e35. 10.1016/j.cell.2020.06.013 32649874 PMC7373300

[B15] Hanahan D. Weinberg R. A. (2011). Hallmarks of Cancer: the Next Generation. Cell 144 (5), 646–674. 10.1016/j.cell.2011.02.013 21376230

[B16] Hashemi S. Fransen M. F. Niemeijer A. Ben Taleb N. Houda I. Veltman J. (2021). Surprising Impact of Stromal TIL's on Immunotherapy Efficacy in a Real-World Lung Cancer Study. Lung Cancer 153, 81–89. 10.1016/j.lungcan.2021.01.013 33465698

[B17] Hensley C. T. Faubert B. Yuan Q. Lev-Cohain N. Jin E. Kim J. (2016). Metabolic Heterogeneity in Human Lung Tumors. Cell 164 (4), 681–694. 10.1016/j.cell.2015.12.034 26853473 PMC4752889

[B18] Huang J. Li J. Zheng S. Lu Z. Che Y. Mao S. (2020a). Tumor Microenvironment Characterization Identifies Two Lung Adenocarcinoma Subtypes with Specific Immune and Metabolic State. Cancer Sci. 111 (6), 1876–1886. 10.1111/cas.14390 32187778 PMC7293093

[B19] Huang Z. Su W. Lu T. Wang Y. Dong Y. Qin Y. (2020b). First-Line Immune-Checkpoint Inhibitors in Non-small Cell Lung Cancer: Current Landscape and Future Progress. Front. Pharmacol. 11, 578091. 10.3389/fphar.2020.578091 33117170 PMC7577011

[B20] Jin R. Liu C. Zheng S. Wang X. Feng X. Li H. (2020). Molecular Heterogeneity of Anti-PD-1/pd-L1 Immunotherapy Efficacy Is Correlated with Tumor Immune Microenvironment in East Asian Patients with Non-small Cell Lung Cancer. Cancer Biol. Med. 17 (3), 768–781. 10.20892/j.issn.2095-3941.2020.0121 32944405 PMC7476088

[B21] Johnson W. E. Li C. Rabinovic A. (2007). Adjusting Batch Effects in Microarray Expression Data Using Empirical Bayes Methods. Biostatistics 8 (1), 118–127. 10.1093/biostatistics/kxj037 16632515

[B22] Kano H. Ichihara E. Harada D. Inoue K. Kayatani H. Hosokawa S. (2020). Utility of Immune Checkpoint Inhibitors in Non‐small‐cell Lung Cancer Patients with Poor Performance Status. Cancer Sci. 111 (10), 3739–3746. 10.1111/cas.14590 32726470 PMC7540975

[B23] Kareva I. Hahnfeldt P. (2013). The Emerging "hallmarks" of Metabolic Reprogramming and Immune Evasion: Distinct or Linked? Cancer Res. 73 (9), 2737–2742. 10.1158/0008-5472.can-12-3696 23423980

[B24] Kim N. Kim H. K. Lee K. Hong Y. Cho J. H. Choi J. W. (2020). Single-cell RNA Sequencing Demonstrates the Molecular and Cellular Reprogramming of Metastatic Lung Adenocarcinoma. Nat. Commun. 11 (1), 2285. 10.1038/s41467-020-16164-1 32385277 PMC7210975

[B25] Lazarou G. Chelliah V. Small B. G. Walker M. Graaf P. H. Kierzek A. M. (2020). Integration of Omics Data Sources to Inform Mechanistic Modeling of Immune‐Oncology Therapies: A Tutorial for Clinical Pharmacologists. Clin. Pharmacol. Ther. 107 (4), 858–870. 10.1002/cpt.1786 31955413 PMC7158209

[B26] Leek J. T. Johnson W. E. Parker H. S. Jaffe A. E. Storey J. D. (2012). The Sva Package for Removing Batch Effects and Other Unwanted Variation in High-Throughput Experiments. Bioinformatics 28 (6), 882–883. 10.1093/bioinformatics/bts034 22257669 PMC3307112

[B27] Leek J. T. Scharpf R. B. Bravo H. C. Simcha D. Langmead B. Johnson W. E. (2010). Tackling the Widespread and Critical Impact of Batch Effects in High-Throughput Data. Nat. Rev. Genet. 11 (10), 733–739. 10.1038/nrg2825 20838408 PMC3880143

[B28] Meyers D. E. Banerji S. (2020). Biomarkers of Immune Checkpoint Inhibitor Efficacy in Cancer. Curr. Oncol. 27 (Suppl. 2), S106–S114. 10.3747/co.27.5549 32368180 PMC7194000

[B29] Miao Y. R. Zhang Q. Lei Q. Luo M. Xie G. Y. Wang H. (2020). ImmuCellAI: A Unique Method for Comprehensive T‐Cell Subsets Abundance Prediction and its Application in Cancer Immunotherapy. Adv. Sci. 7 (7), 1902880. 10.1002/advs.201902880 PMC7141005 32274301

[B30] Park C. Na K. J. Choi H. Ock C.-Y. Ha S. Kim M. (2020). Tumor Immune Profiles Noninvasively Estimated by FDG PET with Deep Learning Correlate with Immunotherapy Response in Lung Adenocarcinoma. Theranostics 10 (23), 10838–10848. 10.7150/thno.50283 32929383 PMC7482798

[B31] Pathak R. Pharaon R. R. Mohanty A. Villaflor V. M. Salgia R. Massarelli E. (2020). Acquired Resistance to PD-1/pd-L1 Blockade in Lung Cancer: Mechanisms and Patterns of Failure. Cancers 12 (12), 3851. 10.3390/cancers12123851 PMC7767234 33419311

[B32] Seo J.-S. Kim A. Shin J.-Y. Kim Y. T. (2018). Comprehensive Analysis of the Tumor Immune Micro-environment in Non-small Cell Lung Cancer for Efficacy of Checkpoint Inhibitor. Sci. Rep. 8 (1), 14576. 10.1038/s41598-018-32855-8 30275546 PMC6167371

[B33] Siegel R. L. Miller K. D. Fuchs H. E. Jemal A. (2021). Cancer Statistics, 2021. CA A. Cancer J. Clin. 71 (1), 7–33. 10.3322/caac.21654 33433946

[B34] Smolle E. Leko P. Stacher‐Priehse E. Brcic L. El‐Heliebi A. Hofmann L. (2020). Distribution and Prognostic Significance of Gluconeogenesis and Glycolysis in Lung Cancer. Mol. Oncol. 14 (11), 2853–2867. 10.1002/1878-0261.12780 32777161 PMC7607181

[B35] Song Y. Yan S. Fan W. Zhang M. Liu W. Lu H. (2020). Identification and Validation of the Immune Subtypes of Lung Adenocarcinoma: Implications for Immunotherapy. Front. Cel Dev. Biol. 8, 550. 10.3389/fcell.2020.00550 PMC7348081 32719796

[B36] Speiser D. E. Ho P.-C. Verdeil G. (2016). Regulatory Circuits of T Cell Function in Cancer. Nat. Rev. Immunol. 16 (10), 599–611. 10.1038/nri.2016.80 27526640

[B37] Sun S. Guo W. Wang Z. Wang X. Zhang G. Zhang H. (2020). Development and Validation of an Immune‐related Prognostic Signature in Lung Adenocarcinoma. Cancer Med. 9 (16), 5960–5975. 10.1002/cam4.3240 32592319 PMC7433810

[B38] Wang Q. Li M. Yang M. Yang Y. Song F. Zhang W. (2020). Analysis of Immune-Related Signatures of Lung Adenocarcinoma Identified Two Distinct Subtypes: Implications for Immune Checkpoint Blockade Therapy. Aging 12 (4), 3312–3339. 10.18632/aging.102814 32091408 PMC7066911

[B39] Wilkerson M. D. Hayes D. N. (2010). ConsensusClusterPlus: a Class Discovery Tool with Confidence Assessments and Item Tracking. Bioinformatics 26 (12), 1572–1573. 10.1093/bioinformatics/btq170 20427518 PMC2881355

[B40] Wu Y. Lin L. Liu X. (2020). Identification of PDL1-Related Biomarkers to Select Lung Adenocarcinoma Patients for PD1/PDL1 Inhibitors. Dis. Markers 2020, 7291586. 10.1155/2020/7291586 32587640 PMC7303743

[B41] Xiao Z. Locasale J. W. Dai Z. (2020). Metabolism in the Tumor Microenvironment: Insights from Single-Cell Analysis. Oncoimmunology 9 (1), 1726556. 10.1080/2162402x.2020.1726556 32117592 PMC7028342

[B42] Xu F. Chen J.-x. Yang X.-b. Hong X.-b. Li Z.-x. Lin L. (2020). Analysis of Lung Adenocarcinoma Subtypes Based on Immune Signatures Identifies Clinical Implications for Cancer Therapy. Mol. Ther. - Oncolytics 17, 241–249. 10.1016/j.omto.2020.03.021 32346613 PMC7183104

[B43] Zhang Y. Yang M. Ng D. M. Haleem M. Yi T. Hu S. (2020). Multi-omics Data Analyses Construct TME and Identify the Immune-Related Prognosis Signatures in Human LUAD. Mol. Ther. - Nucleic Acids 21, 860–873. 10.1016/j.omtn.2020.07.024 32805489 PMC7452010

[B44] Zhao C. Kong X. Han S. Li X. Wu T. Zhou J. (2020). Analysis of Differential Metabolites in Lung Cancer Patients Based on Metabolomics and Bioinformatics. Future Oncol. 16 (18), 1269–1287. 10.2217/fon-2019-0818 32356461

[B45] Zhong R. Chen D. Cao S. Li J. Han B. Zhong H. (2021). Immune Cell Infiltration Features and Related Marker Genes in Lung Cancer Based on Single-Cell RNA-Seq. Clin. Transl Oncol. 23 (2), 405–417. 10.1007/s12094-020-02435-2 32656582

