# Supplementary material for: The Predictive Role of Immune Related Subgroup Classification in Immune Checkpoint Blockade Therapy for Lung Adenocarcinoma
Source: Front Genet. 2021 Oct 15;12:771830. doi: 10.3389/fgene.2021.771830 (PMC8554034; doi:10.3389/fgene.2021.771830)
Supplement: Supplementary file 1 [file DataSheet1.PDF]

The predictive role of immune related subgroup classification in immune checkpoint blockade therapy for lung adenocarcinoma

Xiaozhou Yu[1,2,3#], Ziyang Wang[1,2,3,4#], Yiwen Chen[1,2,3], Guotao Yin[1,2,3], Jianjing Liu[1,2,3], Wei Chen[1,2,3], Lei Zhu[1,2,3], Wengui Xu[1,2,3*], Xiaofeng Li[1,2,3*]

Supplementary materials

Supplementary Figure 1. Validation of immune related subgroup clustering for LUAD from GSE72094 and GSE68465 data sets. **(A)** Consensus matrix of GSE72094 data set when cluster number k=3, and principal component analysis (PCA) plot of clustered samples of GSE72094 data set, where samples in subgroup 1 are marked in red, subgroup 2 in green, and subgroup 3 in blue. **(B)** Consensus matrix of GSE68465 data set when cluster number k=3, and PCA plot of clustered samples of GSE68465 data set, where samples in subgroup 1 are marked in red, subgroup 2 in green, and subgroup 3 in blue.

Supplementary Figure 2. Validation of immune checkpoint associated genes scores in three subgroups using GSE72094 and GSE68465 data sets. **(A)** A heat map of 6 immune checkpoint associated gene scores (CTLA4, CD28, CD80, CD86, CD274, and PDCD1) for LUAD cohort from GSE72094 data set based on immune related subgroup clustering. **(B)** Box plots to present the difference of each of these 6 immune checkpoint associated genes between the identified subgroups for LUAD cohort from GSE72094 data set. (C) A heat map of 6 immune checkpoint associated gene scores (CTLA4, CD28, CD80, CD86, CD274, and PDCD1) for LUAD cohort from GSE68465 data set based on immune associated subgroup clustering. (D) Box plots to present the difference of each of these 6 immune checkpoint associated genes between the identified subgroups for LUAD cohort from GSE68465 data set.

Supplementary Figure 3. Validation of immune cell infiltration scores in three subgroups using GSE72094 and GSE68465 data sets. **(A)** A heat map of immune cell infiltration scores for LUAD cohort from GSE72094 data set based on immune related subgroup clustering. **(B)** Box plots to present the difference of the general infiltration scores, and cell infiltration scores for $CD8^+$ T cells, $CD8^+$ naive T cells, cytotoxic cells, natural regulatory T cells (nTreg), induced regulatory T cells (iTreg) between the identified subgroups of LUAD cohort from GSE72094 data set. **(C)** A heat map of immune cell infiltration scores for LUAD cohort from GSE68465 data set based on immune related subgroup clustering. **(D)** Box plots to present the difference of the general infiltration scores, and cell infiltration scores for $CD8^+$ T cells, $CD8^+$ naive T cells, cytotoxic cells, natural regulatory T cells (nTreg), induced regulatory T cells (iTreg) between the identified subgroups of LUAD cohort from GSE68465 data set.

Supplementary Figure 4. Validation of TIDE analysis and survival analysis for LUAD. **(A)** A heat map of TIDE analysis scores for LUAD cohort from GSE72094 data set based on immune related subgroup clustering. **(B)** Box plots to present the difference in each of the scores from TIDE analysis between the identified subgroups of LUAD cohort from GSE72094 data set. **(C)** Kaplan Meier analysis was performed

to estimate the survival of LUAD cohort based on the immune related subgroup clustering from GSE72094 data set. As shown, subgroup 3 tended to have an unfavorable prognosis in comparison with subgroup 1 and subgroup 2. **(D)** A multivariate Cox proportional hazards regression analysis was performed to identify independent risk factors for LUAD cohort from GSE72094 data set after an univariate Cox proportional hazards regression analysis. As shown in the forest plots, immnune associated subgroup clustering, tumor stage and B cells were suggested as potential independent factors influencing overall survival (OS) of LUAD ($p < 0.05$). GSE68465 data set was not used as validation cohort to perform TIDE analysis and survival analysis because of the lack of information for CD274.

Supplementary Figure 5. Validation of GSEA to identify underlying metabolic mechanism for immune related subgroup clustering of LUAD. Based on the immune related subgroup clustering (subgroup 1 vs subgroup 3), GSEA were performed on LUAD cohort from GSE72094 data set **(A)** and GSE68465 **(B)** data sets using the gene sets significantly associated with glucose metabolism, including process of glycolysis (Normalized Enrichment Score (NES) = 2.29, $P < 0.01$, $Q < 0.05$), gluconeogenesis (NES = 1.74, $P < 0.01$, $Q < 0.05$), tricarboxylic acid (TCA) cycle (NES = 1.86, $P < 0.01$, $Q < 0.05$), oxidative phosphorylation (OXPHOS) (NES = 1.9564092, $P < 0.01$, $Q < 0.05$) and pyruvate metabolism (NES = 1.98, $P < 0.01$, $Q < 0.05$). FDR < 0.05 was the screening threshold. An upward parabola indicated that the indicated process was enhanced in subgroup 1 in contrast with subgroup 3. The barcode plot indicates the position of the genes in each gene set; red and blue colors represent positive and negative Pearson's correlation with subgroup classification (subgroup 1 vs subgroup 3).

Supplementary Figure 1

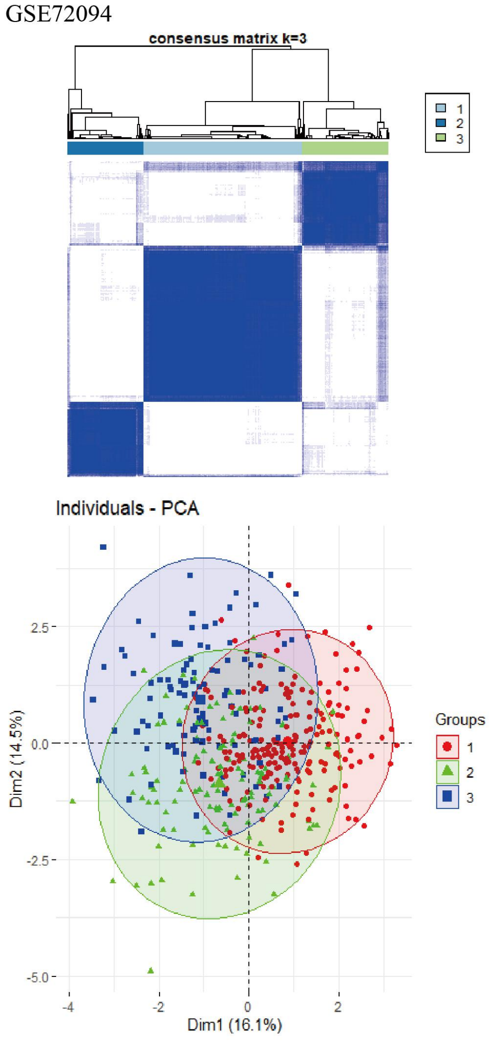

(a)

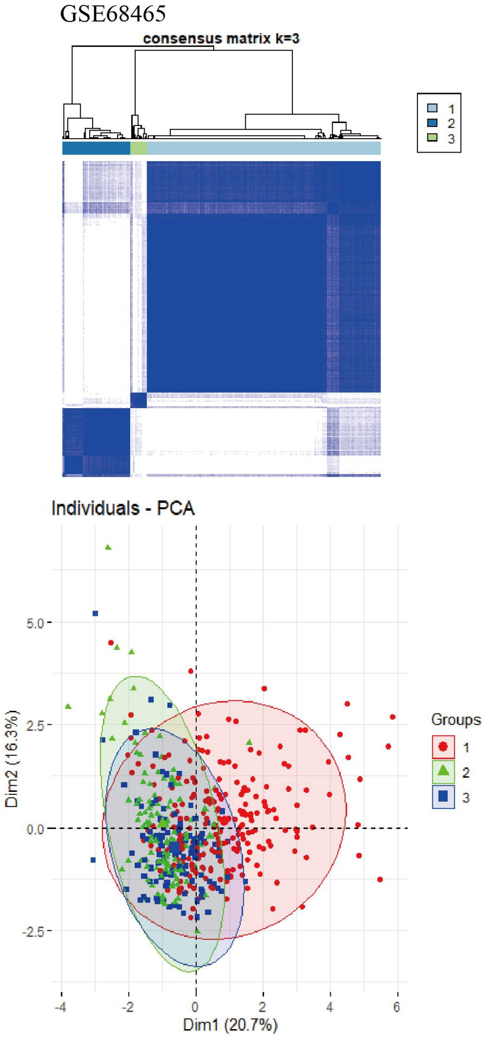

(b)

Supplementary Figure 2

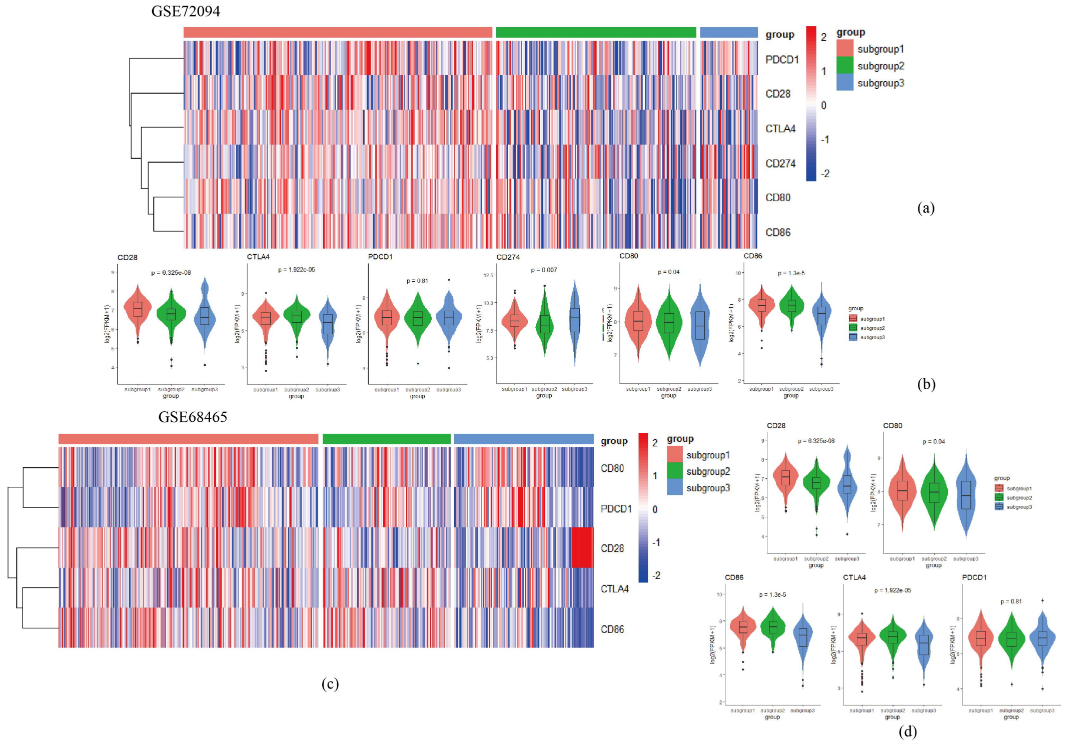

Supplementary Figure 3

GSE72094

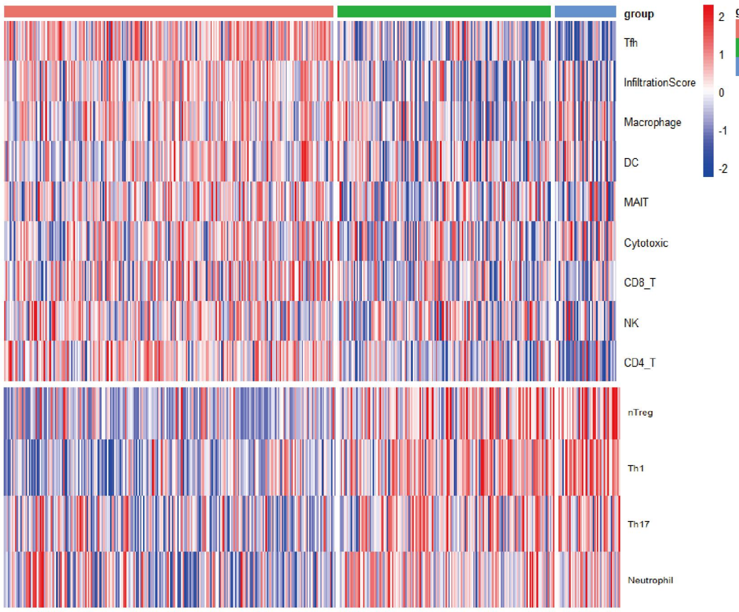

(a)

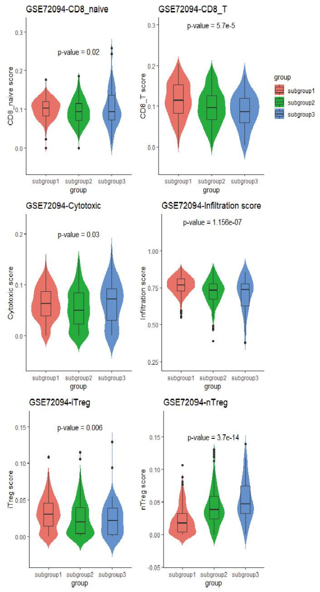

(b)

GSE68465

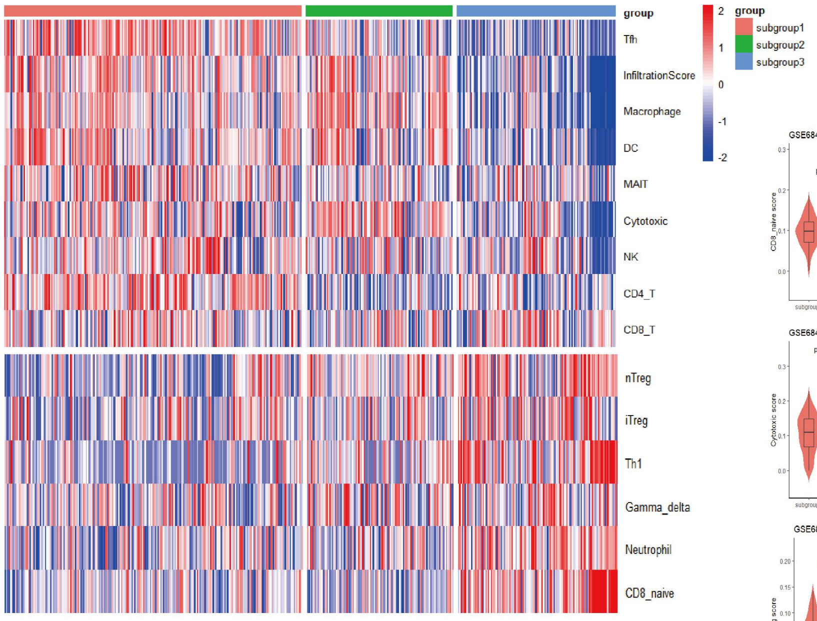

(c)

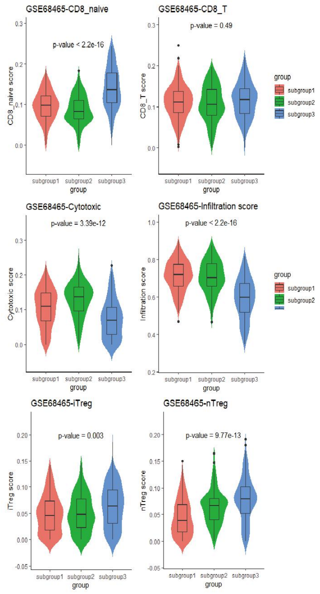

(d)

Supplementary Figure 4

GSE72094

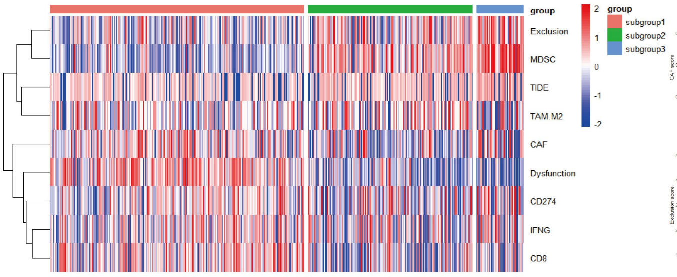

(a)

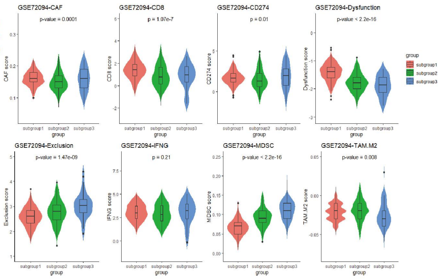

(b)

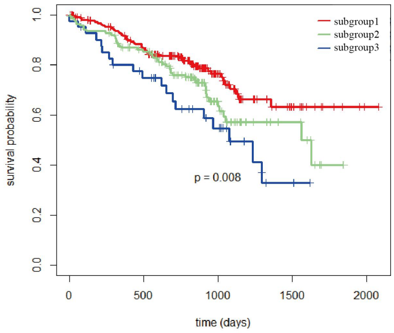

(c)

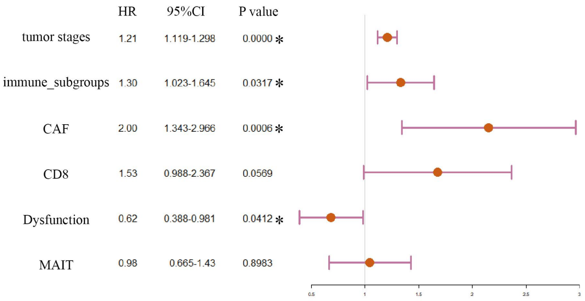

(d)

Supplementary Figure 5

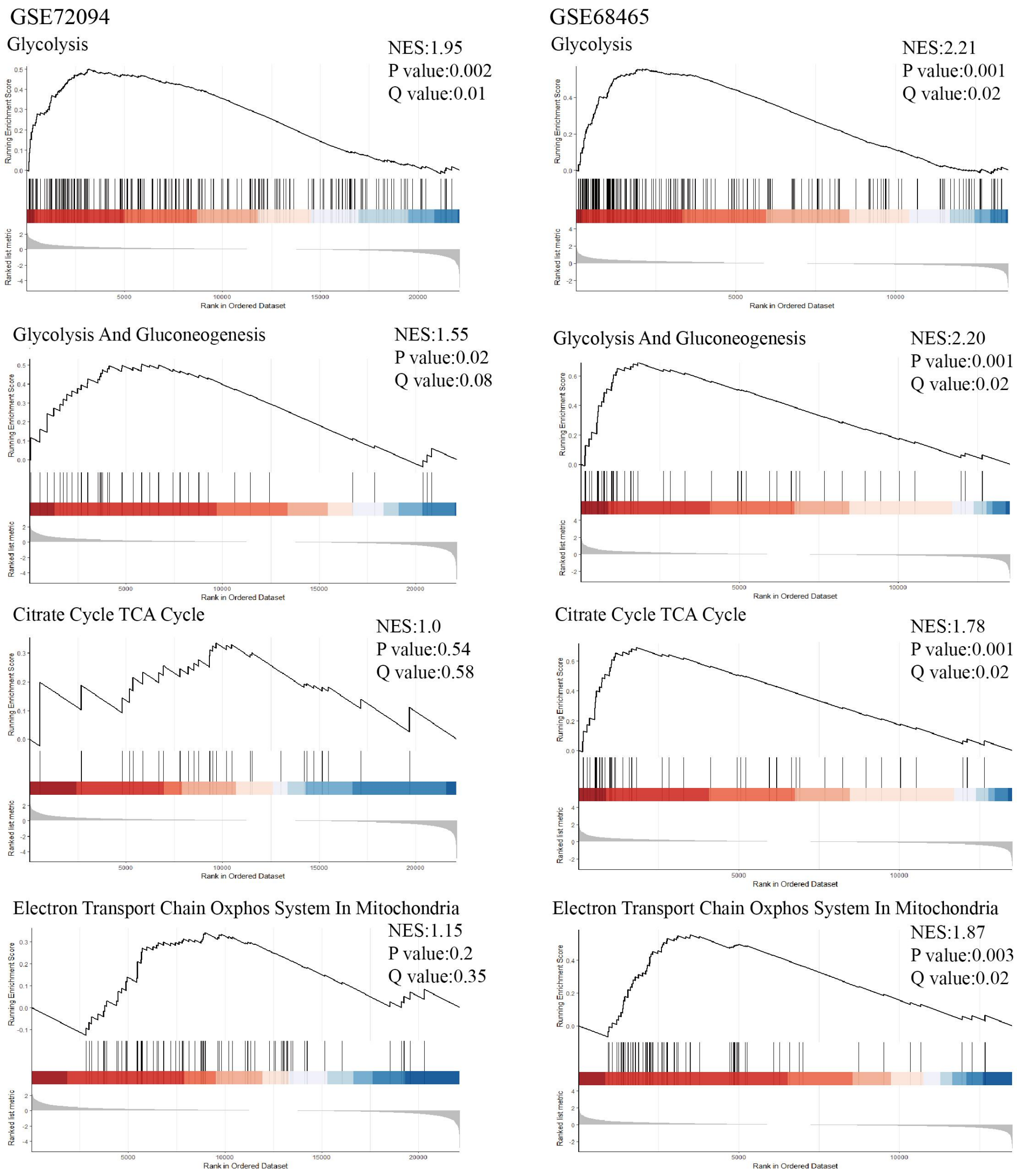

(a) (b)
